# Supplementary material for: Using referral rates for genetic testing to determine the incidence of a rare disease: The minimal incidence of congenital hyperinsulinism in the UK is 1 in 28,389
Source: PLoS One. 2020 Feb 6;15(2):e0228417. doi: 10.1371/journal.pone.0228417 (PMC7004321; doi:10.1371/journal.pone.0228417)
Supplement: S1 Table — (DOCX) [file pone.0228417.s001.docx]

**S1 Table.**

|  |  | **Raw Data** | | **95% CI** | |
| --- | --- | --- | --- | --- | --- |
|  | **Incidence per 100,000** | **Numerator** | **Denominator** | **Lower** | **Upper** |
| **UK** | 3.5 | 278 | 7,892,004 | 3.1 | 4.0 |
| **Ireland** | 3.7 | Unpublished data | |  |  |
| **Netherlands** | 2.0 | 5 | 250,000 | 0.6 | 4.7 |
| **Czech Republic** | 2.2 | 39 | 1,738,168 | 1.6 | 3.1 |
| **Finland** | 2.5 | 24 | 969,600 | 1.6 | 3.7 |
| **Japan** | 2.8 | Data n/a | |  |  |
