# Supplementary material for: Using referral rates for genetic testing to determine the incidence of a rare disease: The minimal incidence of congenital hyperinsulinism in the UK is 1 in 28,389
Source: PLoS One. 2020 Feb 6;15(2):e0228417. doi: 10.1371/journal.pone.0228417 (PMC7004321; doi:10.1371/journal.pone.0228417)
Supplement: S2 Table — The cases of CHI had either persisted beyond 6 months of age or required pancreatectomy for hypoglycaemia. (DOCX) [file pone.0228417.s002.docx]

**S2 Table.**

| **Year** | **Number of Cases of CHI** | **Number of Live Births** | **Annual Incidence** |
| --- | --- | --- | --- |
| 2007 | 28 | 772,245 | 1: 27,580 |
| 2008 | 31 | 794,383 | 1: 25,625 |
| 2009 | 24 | 790,204 | 1: 32,925 |
| 2010 | 28 | 807,271 | 1: 28,831 |
| 2011 | 20 | 807,776 | 1: 40,389 |
| 2012 | 25 | 812,970 | 1: 32,519 |
| 2013 | 25 | 778,803 | 1: 31,152 |
| 2014 | 34 | 776,352 | 1: 22,834 |
| 2015 | 35 | 777,165 | 1: 22,205 |
| 2016 | 28 | 774,835 | 1: 27,673 |
| **Overall** | **278** | **7,892,004** | **1: 28,389** |
