# Supplementary material for: Using referral rates for genetic testing to determine the incidence of a rare disease: The minimal incidence of congenital hyperinsulinism in the UK is 1 in 28,389
Source: PLoS One. 2020 Feb 6;15(2):e0228417. doi: 10.1371/journal.pone.0228417 (PMC7004321; doi:10.1371/journal.pone.0228417)
Supplement: Attachment — Submitted filename: Yau Response to Reviewers_final.docx [file pone.0228417.s003.docx]

Department of Pediatric Endocrinology

Royal University Hospital

University of Saskatchewan

103 Hospital Drive

Saskatoon, Canada S7N 0W8

18 December 2019

Dr Klaus Brusgaard
Academic Editor
PLOS ONE

Dear Dr Brusgaard

Thank you for your reply regarding our manuscript, “Using referral rates for genetic testing to determine the incidence of a rare disease: the minimal incidence of congenital hyperinsulinism in the UK is 1 in 28,389”. We have addressed the points raised during the review process and enclose a point-by-point response as well as revised manuscript. We hope these changes are found to be satisfactory.

Thank you; yours sincerely,

Daphne Yau, MD

**Comments to the Author**

**1. Is the manuscript technically sound, and do the data support the conclusions?**

The manuscript must describe a technically sound piece of scientific research with data that supports the conclusions. Experiments must have been conducted rigorously, with appropriate controls, replication, and sample sizes. The conclusions must be drawn appropriately based on the data presented.

Reviewer #1: Partly

Reviewer #2: Yes

**2. Has the statistical analysis been performed appropriately and rigorously?**

Reviewer #1: No

Reviewer #2: Yes

**3. Have the authors made all data underlying the findings in their manuscript fully available?**

The PLOS Data policy requires authors to make all data underlying the findings described in their manuscript fully available without restriction, with rare exception (please refer to the Data Availability Statement in the manuscript PDF file). The data should be provided as part of the manuscript or its supporting information, or deposited to a public repository. For example, in addition to summary statistics, the data points behind means, medians and variance measures should be available. If there are restrictions on publicly sharing data—e.g. participant privacy or use of data from a third party—those must be specified.

Reviewer #1: No

Reviewer #2: Yes

**The genotype data could be used to identify individuals and so cannot be made openly available. Access to data is open through collaboration. Requests for collaboration will be considered following an application to the Genetic Beta Cell Research Bank (**[**https://www.diabetesgenes.org/current-research/genetic-beta-cell-research-bank/**](https://www.diabetesgenes.org/current-research/genetic-beta-cell-research-bank/)**). Contact by email should be directed to the Lead Nurse, Dr Bridget Knight (**[**b.a.knight@exeter.ac.uk**](mailto:b.a.knight@exeter.ac.uk)**).**

**4. Is the manuscript presented in an intelligible fashion and written in standard English?**

PLOS ONE does not copyedit accepted manuscripts, so the language in submitted articles must be clear, correct, and unambiguous. Any typographical or grammatical errors should be corrected at revision, so please note any specific errors here.

Reviewer #1: Yes

Reviewer #2: Yes

**5. Review Comments to the Author**

Please use the space provided to explain your answers to the questions above. You may also include additional comments for the author, including concerns about dual publication, research ethics, or publication ethics. (Please upload your review as an attachment if it exceeds 20,000 characters)

**Reviewer #1: This is an interesting study of clinical importance, however with some epidemiological flaws.**

1. In the methods, it is not clear how the clinical data were obtained. Did you have access to hospital file data or were the data based on questionnaires in the genetic testing request form? Genetic testing usually takes place prior to surgery. How did you obtain the surgery data? How did you know what age the patients had when determining persistence > 6 or 12 months?

**We thank the Reviewer for their positive and constructive comments.**

**The clinical data were obtained from the genetic testing request form, which encompassed details regarding hyperinsulinism treatment including surgery. If the outcome was unknown, the requesting clinician was contacted to provide data regarding persistence at 6 and 12 months of age, and treatment including surgery. We have revised the manuscript to reflect this.**

2. Methods: An “or” diagnostic criteria of elevated C-peptide is doubtful, as the prolonged half-life of C-peptide compared to insulin implies that p-C-peptide does not reflect rapid changes in p-glucose. Indeed, the two references mentioned (Stanley, Banerjee) did not use elevated C-peptide as a diagnostic criteria. However, the inclusion criteria were sound (>6 mo CHI or surgery). However, it is very unlikely that an infant with an erroneous diagnosis of CHI based on C-peptide would be subjected to pancreatectomy or would still require therapy after 6 mo. The diagnostic problem on C-peptide should be mentioned in Limitations.

**We acknowledge the Reviewer’s point regarding the use of C-peptide in diagnosing hyperinsulinism. We used C-peptide as an additional biomarker in the context of recurrent, severe hypoglycaemia and high glucose infusion rates consistent with hyperinsulinism causing hypoglycaemia while recognizing the importance of insulin as a prime diagnostic marker. We have revised the manuscript to reflect the Reviewer’s comments.**

3. In Results, the only included patients had CHI persisting > 6 months’ age. Did no patient undergo pancreatic surgery <6 months’ age? And if they did, did you miss to detect them?

**We thank the Reviewer for clarifying this. The patients undergoing surgery were included in N=278 patients. We apologise for not having made this clearer and have amended the manuscript to indicate the number of patients undergoing pancreatic surgery.**

4. Why are the p-glucose, p-insulin and C-peptide data not presented? Is the study based on genetic testing requests only without actual data on the diagnosis? If the diagnoses were not actually validated it would be fair to state.

**The genetic testing request form included data on glucose, insulin and c-peptide to confirm evidence of CHI prior to genetic testing. We have now included these values in the results of the manuscript.**

5. A patient inclusion flow chart is missing. The 104 patients lost to follow-up should be presented in a Results flow chart Figure 1. Why was it not possible in a country like the UK to perform a follow-up on these children?

**A patient inclusion flow chart was provided as a supplemental figure. We have now provided this as figure 1 and the incidence data as figure 2. For the 104 patients lost to follow up, we had contacted the referring physicians to obtain outcome data, but either the patient was no longer in the physician’s care or were lost to their follow up, or we did not receive a reply despite several attempts.**

6. An incidence calculation is always an estimate of the true, unknown incidence. Although many publications on incidences do not deal with details, a report on incidence only definitely should. For epidemiologists, a 95% confidence interval would be a demand. Your sampling is large enough to give a narrower C.I. than most others in the field. If you compute 95% C.I.s on the other incidences reported from other countries, one would better understand whether differences are significant or due to small samples.

**We have now calculated 95% confidence intervals for our data set as well as for the incidences reported from other countries. These are included these in figure 2 and the data used for calculation in supporting table S1. However, we were unable to calculate 95% CI for the incidences for Ireland and Japan. The former is based on unpublished data and the latter is published in Japanese.**

**Overall, the estimates from the outbred populations are overlapping or nearly overlapping, in the case of the Czech Republic estimate. It therefore appears the differences are due to small sample sizes in the other populations versus a significant differences between the populations. We have revised our discussion to reflect this analysis.**

7. Moreover, your data set is large enough to dive a little into time trends and geographical distribution. Did the incidence increase with time? This would suggest more referrals rather than a true increase. Were some geographic areas underrepresented? If yes, why?

**As the reviewer has suggested, we have examined the incidence over time (S2 table). Although the annual incidence fluctuated year by year, a sustained increase was not observed over the time period studied. We have not examined the incidence by geography as we do not have data on the location of birth, only location of treatment. It would not be accurate to include data on treatment location given that there are two tertiary referral centres to which patients are referred from all over the UK.**

8. In Discussion, the calculation is presented as a minimal incidence, as probably, or almost, all UK patients had genetic testing done in Exeter. Is it possible that some persistent CHI patients did not undergo genetic testing at all, perhaps especially in the beginning of the period studied?

**We anticipate most if not all patients undergoing genetic testing for CHI in the UK would have been referred to Exeter. However, it is possible, as the reviewer states, that some patients with CHI may not have undergone testing at all, particularly if the hypoglycaemia could be managed with diazoxide.**

**Reviewer #2: This is a well designed and conducted epidemiological study that is presented in a clearly and concisely. The methods and interpretation of data are appropriate. The limitations noted are comprehensive and reasonable. While this is not a landmark study, it does add valuable incidence information that will be useful for planning and operationalizing treatment of a condition that affects a very small population.**

**We kindly thank the Reviewer for their positive comments regarding our study.**
